# RETRACTED: Jiang et al. Measuring Liquid Droplet Size in Two-Phase Nozzle Flow Employing Numerical and Experimental Analyses. *Micromachines* 2022, *13*, 684

**DOI:** 10.3390/mi17060636

**Published:** 2026-05-22

**Authors:** Lin Jiang, Wei Rao, Lei Deng, Atilla Incecik, Grzegorz Królczyk, Zhixiong Li

**Affiliations:** 1 Longyan Tobacco Industry Co., Ltd., Longyan 364021, China; jl22876@fjtic.cn (L.J.); rw20590@fjtic.cn (W.R.); 2 School of Electrical, Computer & Telecommunications Engineering, University of Wollongong, Wollongong, NSW 2522, Australia; leideng@uow.edu.au; 3 Department of Naval Architecture, Ocean, and Marine Engineering, University of Strathclyde, Glasgow G11XQ, UK; atilla.incecik@strath.ac.uk; 4 Department of Manufacturing Engineering and Automation Products, Opole University of Technology, 45-758 Opole, Poland; g.krolczyk@po.opole.pl; 5 Yonsei Frontier Lab, Yonsei University, Seoul 03722, Republic of Korea

The journal retracts the article titled “Measuring Liquid Droplet Size in Two-Phase Nozzle Flow Employing Numerical and Experimental Analyses” [1], cited above. 

Following publication, concerns were brought to the attention of the publisher regarding a potential overlap between this publication [1] and a previously published article [2], with one author in common. 

In accordance with standard journal procedures, an investigation was conducted by the Editorial Office and Editorial Board that confirmed a significant level of overlap, in terms of images, methods and findings, between the above-mentioned publications, without appropriate acknowledgement, citation, or permission. As a result, the Editorial Board has decided to retract this publication in accordance with MDPI’s retraction policy.

This retraction was approved by the Section Editor-in-Chief of the journal *Micromachines*.

The authors have been informed of this retraction and have indicated their disagreement with this decision.
